# Series of periodic limb movements in sleep and heart rate variability

**DOI:** 10.1192/j.eurpsy.2023.608

**Published:** 2023-07-19

**Authors:** M. A. Małkiewicz, M. Grzywinska, E. Eemil Partinen, M. Partinen, W. J. Cubala, P. J. Winklewski, M. Sieminski

**Affiliations:** ^1^Department of Psychiatry; ^2^Applied Cognitive Neuroscience Lab, Department of Human Physiology; ^3^Neuroinformatics and Artificial Intelligence Lab, Department of Human Physiology, Medical University of Gdansk, Gdansk, Poland; ^4^Department of Neurology, Helsinki University Central Hospital; ^5^Department of Neurosciences, Clinicum, University of Helsinki, Helsinki, Finland; ^6^Department of Emergency Medicine, Medical University of Gdansk, Gdansk, Poland

## Abstract

**Introduction:**

The relationship between a series of periodic limb movements in sleep (PLMS) and heart rate variability (HRV) is not clearly established. HRV reflects changes in heart rate (HR) and autonomic nervous system (ANS) tonus as 2 main components: low-frequency HRV (HRV LF) and high-frequency HRV (HRV HF). An accumulating body of evidence suggests that a single PLMS alters HRV parameters. However, less is known about the impact of HRV changes when a series of PLMS occurs. Both PLMS and HRV have been reported to show associations with cardiovascular and psychiatric diseases.

**Objectives:**

PLMS have been associated with diverse psychiatric diseases in numerous studies. Longitudinal data demonstrate that patients with PLMS have an increased risk of depression and anxiety, and dementia. It should be noted, however, that it remains unclear how a series of PLMS affects the autonomic nervous system, cardiovascular system, and mental health. Therefore, the aim of this study was to verify the hypothesis that a series of PLMS is connected with a higher range of abnormalities in systolic and diastolic blood pressure (SBP and DBP, respectively), with particular interest in HRV HF and HRV LF scores before the series of PLMS and after the end of the series.

**Methods:**

We undertook a retrospective analysis of polysomnography (PSG) and demographic and medical data of 5 patients with a total number of 1348 PLMS. We analyzed HR, HRV LF, HRV HF, systolic blood pressure (SBP), and diastolic blood pressure (DBP) for 10 heartbeats before the series of PLMS and 10 consecutive heartbeats as beat-to-beat measurements. The stage of sleep and duration of limb movement in each PLMS were also assessed. Statistics analysis was performed using IBM SPSS Statistics (v28.0.0.0). The Kruskal–Wallis test was performed to find statistically significant changes from the baseline.

**Results:**

No statistically significant changes in HR, SBP, or DBP were found in our group. HRV changed after the series of 8 PLMS, with both HRV HF and HRV LF increasing.

**Conclusions:**

Our study presents for the first time the co-activation of both HRV HF and HRV LF, pointing toward the possible autonomic dysregulation in patients with a series of PLMS.

**Disclosure of Interest:**

None Declared

